# Correction: Shin et al. *miR-29b* Regulates TGF-β1-Induced Epithelial–Mesenchymal Transition by Inhibiting Heat Shock Protein 47 Expression in Airway Epithelial Cells. *Int. J. Mol. Sci.* 2021, *22*, 11535

**DOI:** 10.3390/ijms262411865

**Published:** 2025-12-09

**Authors:** Jae-Min Shin, Joo-Hoo Park, Hyun-Woo Yang, Jee Won Moon, Heung-Man Lee, Il-Ho Park

**Affiliations:** 1 Department of Otorhinolaryngology-Head and Neck Surgery, Korea University College of Medicine, Seoul 08308, Republic of Korea; shinjm0601@hanmail.net (J.-M.S.); aka_yc@naver.com (J.W.M.); lhman@korea.ac.kr (H.-M.L.); 2 Upper Airway Chronic Inflammatory Diseases Laboratory, Korea University College of Medicine, Seoul 08308, Republic of Korea; pjh52763@korea.ac.kr (J.-H.P.); yhw444@korea.ac.kr (H.-W.Y.); 3 Medical Device Usability Test Center, Korea University Guro Hospital, Seoul 08308, Republic of Korea

In the original publication [1], there were errors in the +*miR-29b* mimic (α-SMA) in Figure 2f and +*miR-29b* (α-SMA) Control in Figure 3f; they were repeated. It appears that the α-SMA and E-cad data in Figure 2 were mistakenly left as temporary data and should have been updated. However, only the HSP data was corrected, while the α-SMA data was inadvertently not updated. And the labels in Figures 2c and 3c are incorrect. Figure 2c should be labeled as *miR-29b* mimic. Figure 3c should be labeled as *miR-29b* inhibitor. The corrected Figure 2c,f and Figure 3c appear below. The authors state that the scientific conclusions are unaffected. This correction was approved by the Academic Editor. The original publication has also been updated.

## Figures and Tables

**Figure 2:**
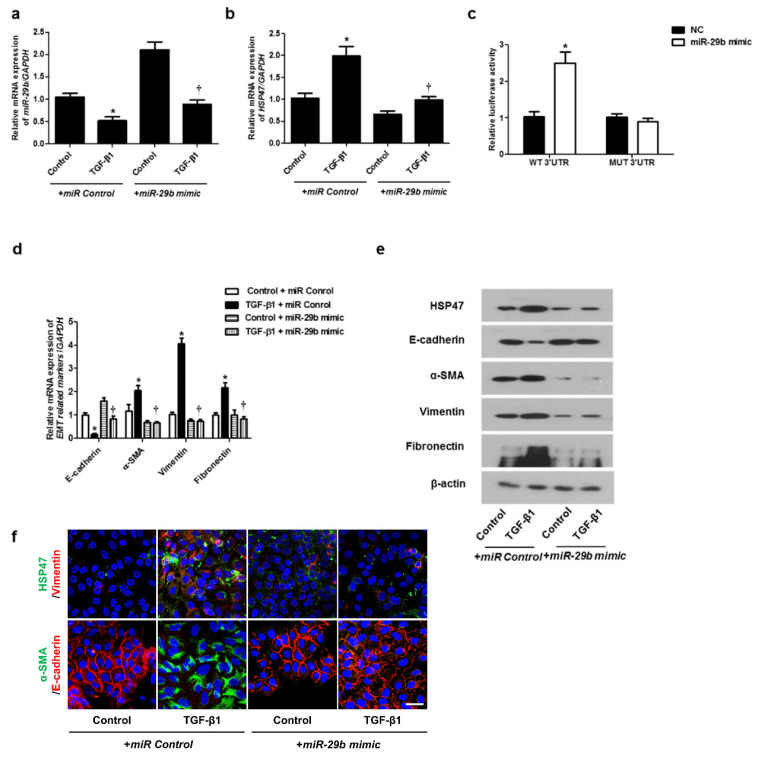
Overexpression of *miR-29b* inhibited mRNA and protein expression levels of TGF-β1-induced EMT markers in A549 cells. A549 cells were stimulated with TGF-β1 (5 ng/mL) with *miR control* or *miR-29b mimic*. (**a**,**b**) mRNA expression levels of *miR-29b* and HSP47 were determined using qPCR. (**c**) HSP47 luciferase activity was measured by luciferase assay. (**d**) *E-cadherin*, *α-SMA*, *vimentin*, and *fibronectin* mRNA levels were analyzed through qPCR. (**e**) Protein expression levels of HSP47, E-cadherin, α-SMA, vimentin, and fibronectin were determined using Western blotting. (**f**) The cells were treated with TGF-β1 for 72 h after transfection of *miR-29b mimic* and then assessed for HSP47 (1st line, green), vimentin (1st line, red), α-SMA (2nd, green), and E-cadherin (2nd, red) expression/localization using immunofluorescence. Nuclei were stained with DAPI (blue). Scale bar = 20 μm. Values are expressed as mean ± SEM of three independent samples. * *p* < 0.05, vs. control + *miR Control*; † *p* < 0.05, vs. TGF-β1 + *miR Control*.

**Figure 3:**
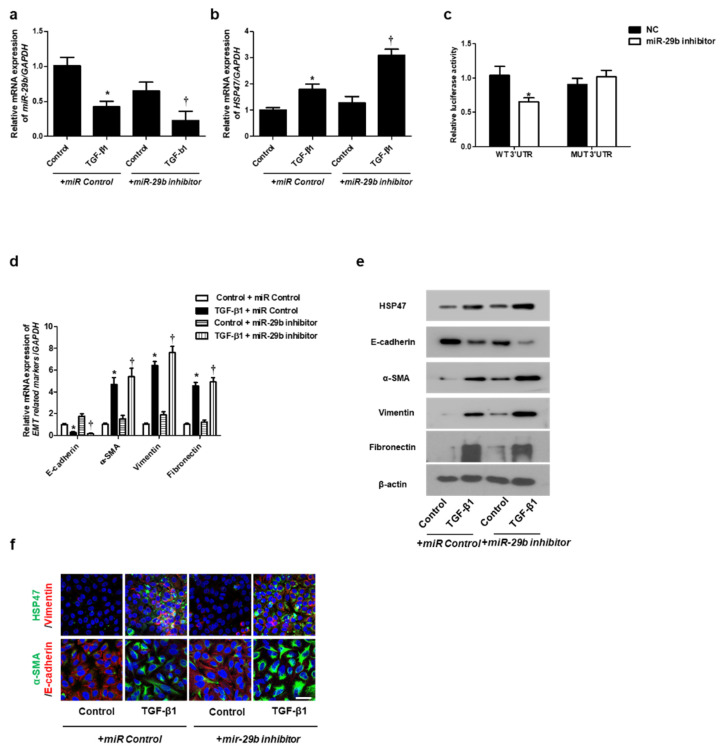
Inhibition of *miR-29b* expression induced mRNA and protein expression levels of TGF-β1-induced EMT markers in A549 cells. A549 cells were stimulated with TGF-β1 (5 ng/mL) with *miR control* or *a miR-29b inhibitor*. (**a**,**b**) The mRNA expression levels of *miR-29b* and *HSP47* were analyzed using qPCR. (**c**) HSP47 luciferase activity was measured by luciferase assay. (**d**) The mRNA levels of *EMT-related markers* were measured using qPCR. (**e**) Protein expression levels of HSP47, E-cadherin, α-SMA, vimentin and fibronectin were determined using Western blotting. (**f**) The cells were treated with TGF-β1 for 72 h after transfection with *miR-29b inhibitor*, and then assessed for HSP47 (1st line, green), vimentin (1st line, red), α-SMA (2nd, green), and E-cadherin (2nd, red) expression/localization using immunofluorescence. Nuclei were stained with DAPI (blue). Scale bar = 20 μm. Values are expressed as mean ± SEM of three independent samples. * *p* < 0.05 vs. control + *miR Control*; † *p* < 0.05, vs. TGF-β1 + *miR Control*.
